# Intermittent Hypoxia Promotes TAM-Induced Glycolysis in Laryngeal Cancer Cells via Regulation of HK1 Expression through Activation of ZBTB10

**DOI:** 10.3390/ijms241914808

**Published:** 2023-09-30

**Authors:** Minlan Yang, Weisong Cai, Zehua Lin, Aikebaier Tuohuti, Xiong Chen

**Affiliations:** Department of Otorhinolaryngology, Head and Neck Surgery, Zhongnan Hospital of Wuhan University, Wuhan 430072, China

**Keywords:** intermittent hypoxia, TAM, HK1, ZBTB10, laryngeal cancer

## Abstract

Obstructive sleep apnea (OSA), characterized by intermittent hypoxia (IH), may increase the risk of cancer development and a poor cancer prognosis. TAMs of the M2 phenotype, together with the intermittent hypoxic environment within the tumor, drive tumor aggressiveness. However, the mechanism of TAMs in IH remains unclear. In our study, IH induced the recruitment of macrophages, and IH-induced M2-like TAMs promoted glycolysis in laryngeal cancer cells through hexokinase 1. The hexokinase inhibitor 2-deoxy-D-glucose and HK1 shRNA were applied to verify this finding, confirming that M2-like TAMs enhanced glycolysis in laryngeal cancer cells through HK1 under intermittent hypoxic conditions. Comprehensive RNA-seq analysis disclosed a marked elevation in the expression levels of the transcription factor ZBTB10, while evaluation of a laryngeal cancer patient tissue microarray demonstrated a positive correlation between ZBTB10 and HK1 expression in laryngeal carcinoma. Knockdown of ZBTB10 decreased HK1 expression, and overexpression of ZBTB10 increased HK1 expression in both laryngeal cancer cells and 293T cells. The luciferase reporter assay and Chromatin immunoprecipitation assay confirmed that ZBTB10 directly bound to the promoter region of HK1 and regulated the transcriptional activity of HK1. Finally, the CLEC3B level of the M2 supernatant is significantly higher in the IH group and showed a protumor effect on Hep2 cells. As ZBTB10-mediated regulation of HK1 affects glycolysis in laryngeal cancer, our findings may provide new potential therapeutic targets for laryngeal cancer.

## 1. Introduction

Obstructive sleep apnea (OSA) is the most common form of sleep apnea and is characterized by recurrent partial or complete obstruction of the upper respiratory tract during sleep [1,2]. It has been estimated that 936 million adults worldwide aged 30–69 years suffer from OSA. Epidemiological evidence suggests that cancer rates are higher in patients with OSA [3]. According to a large multicenter retrospective cohort study, tumor incidence was found to be independently associated with OSA [4,5,6]. Laryngeal cancer is a common malignant tumor of the head and neck, accounting for approximately 1–5% of systemic tumors. Moreover, the study also indicated an association between OSA incidence and head and neck cancer [7]. In patients with laryngeal cancer, the anatomy of the larynx and pharynx is changed through laryngeal function preservation surgery, which can easily lead to the occurrence of OSA [7,8]. However, the mechanism by which OSA affects laryngeal cancer is still unclear.

Intermittent hypoxia, an important clinical feature of patients with OSA, is characterized by repeated hypoxia and reoxygenation [9]. Emerging evidence indicates that IH modulates the host immune response in murine models by exerting a specific influence on tumor-associated macrophages (TAMs) [10,11,12]. TAMs are macrophages that infiltrate tumor tissue and play a protumor and immunosuppressive role in a variety of ways [13]. Activated macrophages mainly include M1 macrophages and M2 macrophages. Most of the macrophages in tumor tissues have the phenotype and function of M2 macrophages; therefore, specific M2 morphological macrophages that exert immunosuppressive and protumor effects are narrowly defined as TAMs [14,15]. Intermittent hypoxia promotes a decrease in tumor suppressor (M1 phenotype) TAMs and a shift to the protumor M2 phenotype [16]. Therefore, IH may influence tumor development by regulating macrophage polarization. However, how macrophages affect laryngeal cancer cells under intermittent hypoxia has not been clarified.

TAMs play a protumor and immunosuppressive role in a variety of ways. TAMs promote the proliferation of tumor cells by secreting EGF, PDGF, etc. [17]. TAMs participate in tumor invasion and metastasis by releasing tumor-promoting factors such as MMPs. TAMs are involved in the immune escape of tumor cells by producing IL10, PGE2, and TGFβ [18]. TAMs-expressed VEGF contributes to the proliferation of both the tumor microvasculature and lymphatic vessels [19]. In addition, TAMs promote tumor growth by regulating tumor cell metabolism, and TAMs have been reported to promote aerobic glycolysis, cell proliferation and tumor growth in glioma, thereby promoting malignant tumor progression [16]. Under hypoxia and lactic acid stimulation, TAMs can also secrete IL-6, CCL5, CCL18, and other cytokines to promote the glycolysis of tumor cells [20,21].

Glycolysis is one of the two main metabolic pathways of cells by which biochemical energy from nutrients is converted into ATP to support essential cellular functions. In addition to providing cellular energy, metabolites of glycolysis play a key role in macromolecular biosynthesis and acidic tumor microenvironment construction, thus giving cancer cells a selective advantage in the face of reduced nutrient supply [22,23,24]. The first step of glycolysis is glucose phosphorylation to generate glucose 6-phosphate. Hexokinase plays an important role in this reaction. Hexokinase has five subtypes, among which subtypes I and II are distributed in the outer membrane of mitochondria and bind to voltage-dependent cation channel proteins, which allow hexokinase to directly bind to ATP produced by mitochondria. The mitochondrial hexokinase is found to be significantly increased in cancer cells [25]. High levels of hexokinase in cancer cells are thought to drive glycolysis to maintain aerobic metabolism in cells, which constitutes the Warburg effect [26]. Based on the TGCA database, a novel hypoxia-related signature for prognostic and immunogenic evaluation in head and neck squamous cell carcinoma containing HK1 was established [27]. In this study, we aimed to explore the effect of TAM on laryngeal cancer with OSA, and investigated the role of HK1 in TAM-inducing glycolysis of laryngeal cancer cells when exposed to IH conditions. We provide evidence that HK1 modulates the glycolytic pathway in TAM exposed to IH. Additionally, we demonstrate that HK1 is transcriptionally regulated by ZBTB10, indicating that targeting ZBTB10 or HK1 may be a potential approach for treating laryngeal cancer patients with OSA.

## 2. Results

**Figure 1:**
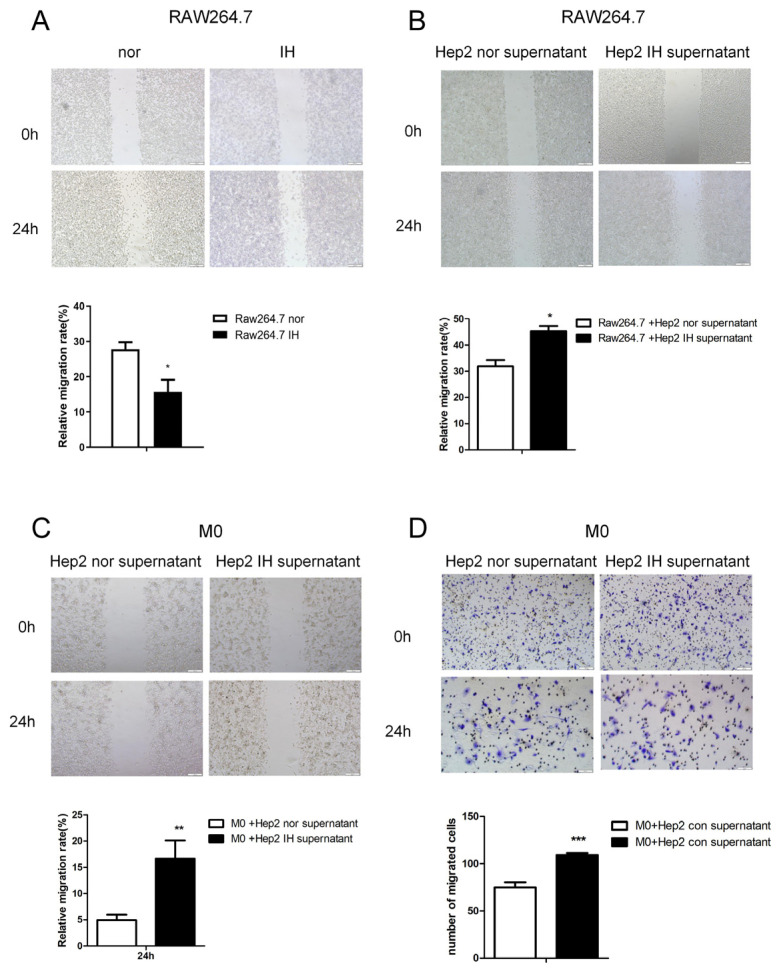
The supernatant of laryngeal cancer cells exposed to IH promoted macrophage migration. (**A**) Wound-healing assay of Raw264.7 cells in a normal or IH microenvironment (100×). (**B**) Wound-healing assay of Raw264.7 cells treated with Hep2 cell culture supernatant in a normal or IH microenvironment (100×). (**C**) Wound-healing assay of M0 macrophages treated with Hep2 cell culture supernatant in a normal or IH microenvironment(100×). (**D**) Transwell migration assays were conducted in M0 macrophages treated with Hep2 cell culture supernatant in a normal or IH microenvironment (100× and 200×). The data are presented as the mean ± SD values. * *p* < 0.05; ** *p* < 0.01; *** *p* < 0.001.

**Figure 2:**
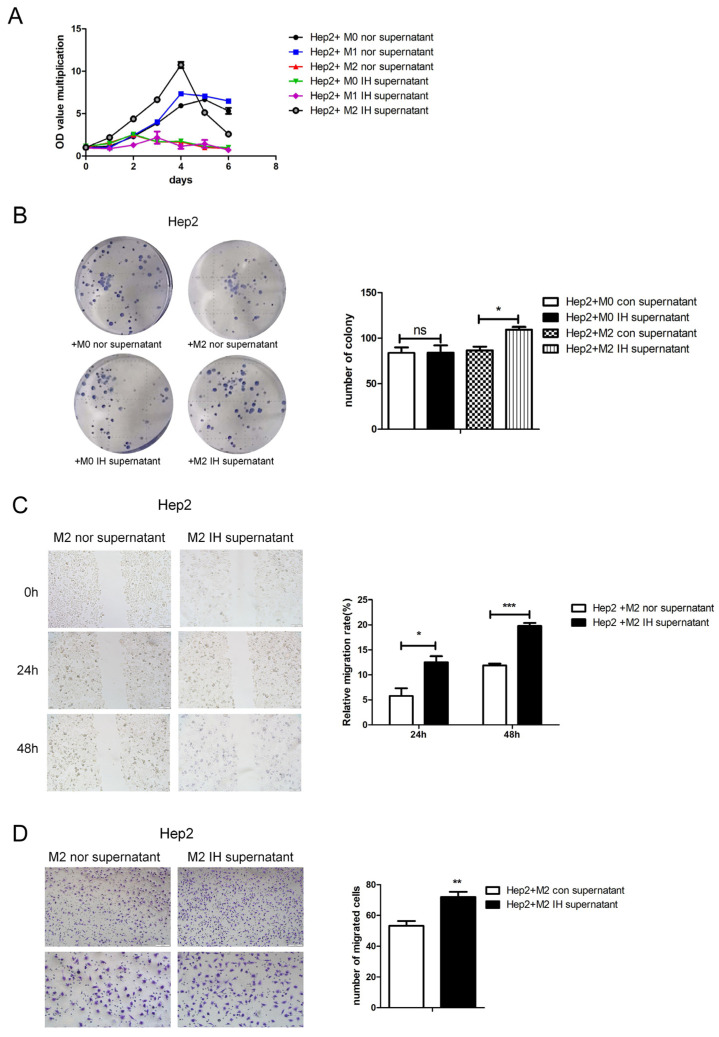
Culture supernatant from M2-type macrophages exposed to intermittent hypoxia increased the growth, proliferation and migration of Hep2 cells. Hep2 cells were treated with normal or IH-exposed M2-type macrophage supernatant, and the following assays were performed. (**A**) CCK8 assay showing the relative proliferation ability. (**B**) Representative images of the wells of 6-well plates in the colony formation assay; the statistical analysis is shown below. (**C**) Wound-healing assay (100×). (**D**) Transwell migration assay in Hep2 cells (100× and 200×). The data are presented as the mean ± SD values. ^ns^ *p* > 0.05; * *p* < 0.05; ** *p* < 0.01; *** *p* < 0.001.

Firstly, we studied the effect of IH on macrophage migration. The wound-healing assay showed that IH inhibited Raw264.7 macrophage cell migration (Figure 1A). The supernatant of laryngeal cancer cells exposed to intermittent hypoxia for 24 h was found to promote RAW264.7 cell migration (Figure 1B–D). This indicates that IH can inhibit cell migration. However, IH may induce laryngeal cancer cells to secrete macrophage aggregation-related factors. Macrophages were induced to polarize toward the M2 type and exposed to intermittent hypoxia for 24 h, and the supernatant was collected and added to laryngeal cancer cells. It was found that culture supernatant from M2-type macrophages exposed to IH promoted malignant phenotypes of laryngeal cancer cells, as indicated by the significant increases in cell growth, proliferation, and migration (Figure 2).

**Figure 3:**
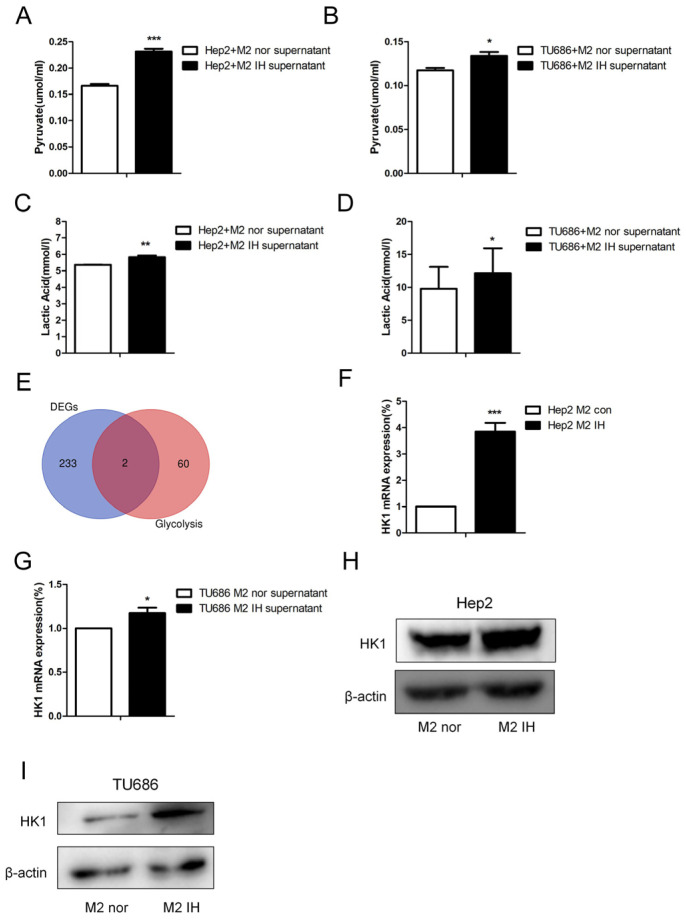
Intermittent hypoxia-exposed M2-type macrophage supernatant enhanced glycolysis and upregulated HK1 expression. (**A**–**D**) The lactate and pyruvate contents of laryngeal carcinoma cells were measured. (**E**) The intersection of DEGs and glycolysis-related genes is shown in a Venn diagram. (**F**,**G**) The HK1 mRNA level in laryngeal carcinoma cells treated with normal or IH-exposed M2-type macrophage supernatant was measured by qPCR. (**H**,**I**) Representative Western blot images showing HK1 protein levels. The data are presented as the mean ± SD values. * *p* < 0.05; ** *p* < 0.01; *** *p* < 0.001.

**Figure 4:**
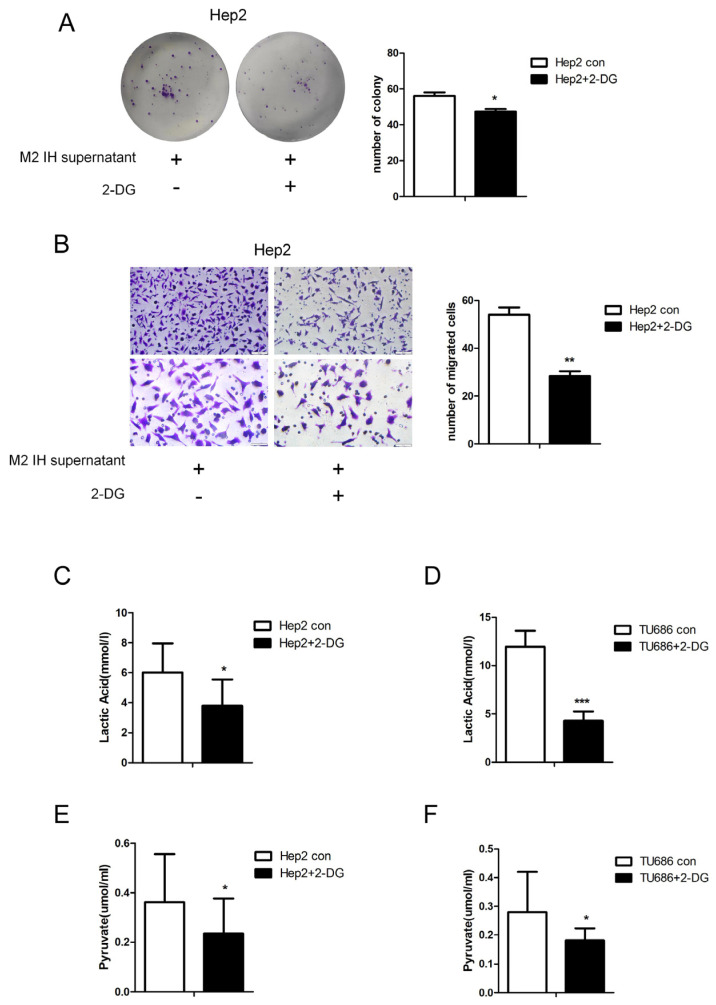
Treatment with the hexokinase inhibitor 2-DG attenuated the proliferation and migration of Hep2 cells induced by intermittent hypoxia-exposed M2-type macrophage supernatant. Hep2 cells were cultured in intermittent hypoxia-exposed M2-type macrophage supernatant and treated with 2-DG or solvent control, and the following assays were performed. (**A**) Colony formation assay. (**B**) Transwell migration assay (100×). (**C**,**D**) Lactate assay. (**E**,**F**) Pyruvate assay. The data are presented as the mean ± SD values. * *p* < 0.05; ** *p* < 0.01; *** *p* < 0.001.

**Figure 5:**
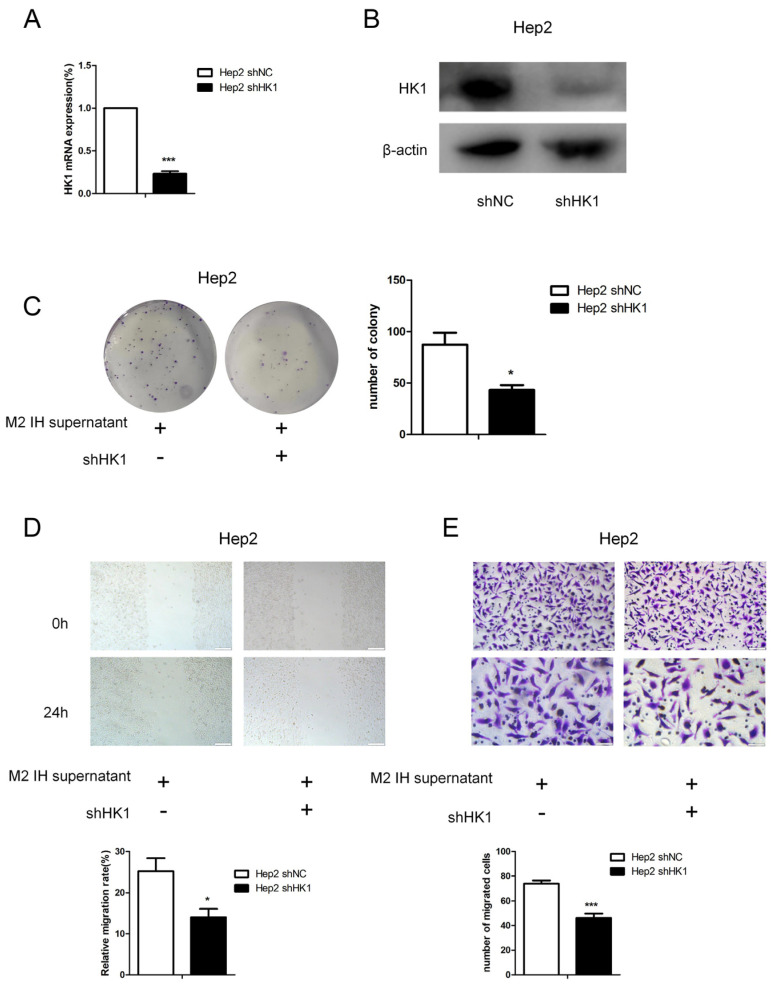
HK1 knockdown attenuated the proliferation and migration of Hep2 cells induced by intermittent hypoxia-exposed M2-type macrophage supernatant. (**A**,**B**) The results of qPCR and Western blotting confirmed HK1 knockdown in Hep2 cells. (**C**) Colony formation assay showing the proliferation of Hep2 cells transfected with shHK1 or shNC. (**D**,**E**) Wound-healing and Transwell migration assays were conducted in Hep2 cells transfected with shHK1 or shNC (100× and 200×). The data are presented as the mean ± SD values. * *p* < 0.05; *** *p* < 0.001.

**Figure 6:**
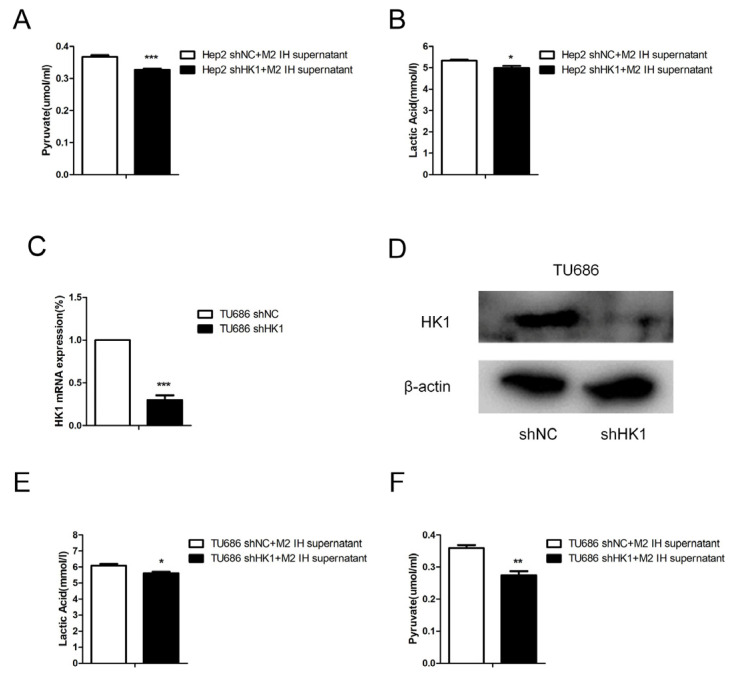
HK1 knockdown attenuated glycolysis induced by intermittent hypoxia-exposed M2-type macrophage supernatant. (**A**,**B**) Lactate and pyruvate assays were conducted in Hep2 cells transfected with shHK1 or shNC and incubated with culture supernatant from intermittent hypoxia-exposed M2-type macrophages. (**C**,**D**) The results of qPCR and Western blot confirmed HK1 knockdown in TU686 cells. (**E**,**F**) Lactate and pyruvate assays were conducted in TU686 cells transfected with shHK1 or shNC and incubated with culture supernatant from intermittent hypoxia-exposed M2-type macrophages. The data are presented as the mean ± SD values. * *p* < 0.05; ** *p* < 0.01; *** *p* < 0.001.

To study the effect of intermittent hypoxia-exposed M2 macrophage supernatant on glycolysis, products of glycolysis, such as lactate and pyruvate, were quantified. After intermittent hypoxia-exposed M2 macrophage supernatant was added to laryngeal cancer cells, the lactate and pyruvate contents in the laryngeal cells were increased. The same results were obtained in both Hep2 and TU686 cells (Figure 3A–D). RNA-seq was used to screen for differentially expressed genes (DEGs), and 235 DEGs were identified. Through analysis of genes related to glycolysis, we found that, in laryngeal cancer cells exposed to M2 macrophage culture supernatant, hexokinase (HK1) expression was elevated. qPCR and Western blot analysis revealed that the mRNA and protein expression levels of HK1 in Hep2 and TU686 cells were elevated, consistent with the sequencing results (Figure 3E–I). Upon treatment with the hexokinase inhibitor 2-DG, it was found that 2-DG reversed the protumor effect of M2 macrophage supernatant on laryngeal cancer cells (Figure 4A,B). The 2-DG treatment also reduced the lactate and pyruvate contents in laryngeal cancer cells (Figure 4C–F). In addition, we constructed HK1-shRNA, which specifically inhibited HK1, and found that silencing HK1 inhibited the proliferation and migration of Hep2 laryngeal cancer cells (Figure 5A–E). Silencing HK1 also reversed the reductions in the lactate and pyruvate contents, which were increased by coculture with M2 macrophage supernatant (Figure 6A,B). The same results were found in TU686 cells when HK1 was silenced (Figure 6C–F).

**Figure 7:**
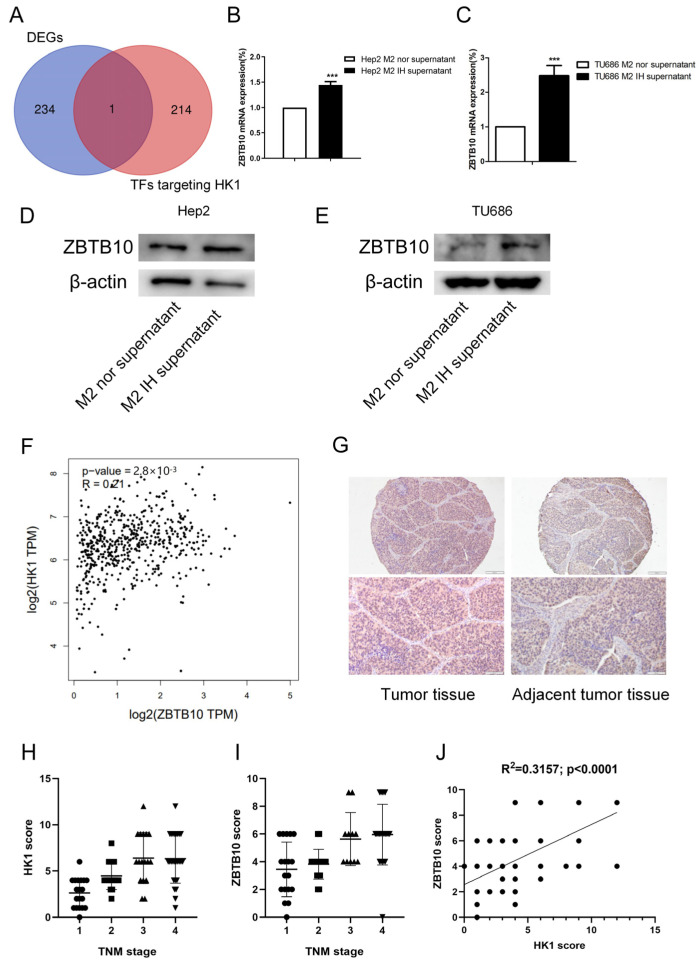
ZBTB10 was identified by screening as the transcription factor targeting HK1. (**A**) The intersection of DEGs and transcription factors targeting HK1 is shown in a Venn diagram. (**B**–**E**) The results of qPCR and Western blotting confirmed the upregulation of ZBTB10 induced by intermittent hypoxia-exposed M2-type macrophage supernatant. (**F**) Correlation analysis between HK1 and ZBTB10 expression based on TCGA data. (**G**) Immunohistochemistry of HK1 and ZBTB10 in 80 samples of human laryngeal cancer tissue (100× and 200×). (**H**,**I**) Kruskal–Wallis one-way analysis of variance by ranks test to determine the correlations between TNM stage and HK1 and ZBTB10 expression. (**J**) Pearson correlation analysis between HK1 and ZBTB10 expression in 80 samples of human laryngeal cancer tissue. The data are presented as the mean ± SD values. *** *p* < 0.001.

To investigate the potential mechanism of HK1, the GeneCards online database (https://www.genecards.org/, accessed on 3 August 2022) was used to search for transcription factors targeting HK1. Through the intersection analysis of transcription factors targeting HK1 and DEGs, ZBTB10 was screened out as the differently expressed transcription factor targeting HK1 (Figure 7A). It has been reported that ZBTB10 is involved in pyruvate kinase metabolism, which is a key process of the glycolysis response essential for cancer progression [28]. The RNA-seq results also revealed that the expression of the transcription factor ZBTB10 was elevated, and we confirmed that ZBTB10 mRNA and protein expression levels were elevated in both Hep2 and TU686 cells treated with conditioned medium, consistent with the RNA-seq results (Figure 7B–E). Then, after analysis with the GEPIA2 online database (http://gepia2.cancer-pku.cn/#correlation, accessed on 7 August 2022), we found that the expression of HK1 and ZBTB10 was positively correlated in head and neck tumors in data from the TCGA database (R = 0.21; *p* < 0.0001) (Figure 7F). We performed immunohistochemical staining of a tissue microarray containing 80 samples of human laryngeal cancer tissue to determine the expression patterns of HK1 and ZBTB10 (Table S1). We found that HK1 was highly expressed in laryngeal cancer tissues and was predominantly localized in the cytoplasm. ZBTB10 was highly expressed in laryngeal cancer tissues and localized in both the nucleus and cytoplasm (Figure 7G). Kruskal–Wallis one-way analysis of variance by ranks test revealed that the expression of HK1 and ZBTB10 was positively correlated with the TNM stage of tumors, i.e., higher grades were correlated with higher expression of HK1 and ZBTB10 (P_HK1_ < 0.001; P_ZBTB10_ = 0.0001 < 0.001) (Figure 7H,I). In addition, Pearson correlation analysis showed that the HK1 expression level was positively correlated with the ZBTB10 expression level in the laryngeal cancer tissue microarray (*R^2^* = 0.3157; *p* < 0.0001) (Figure 7J).

**Figure 8:**
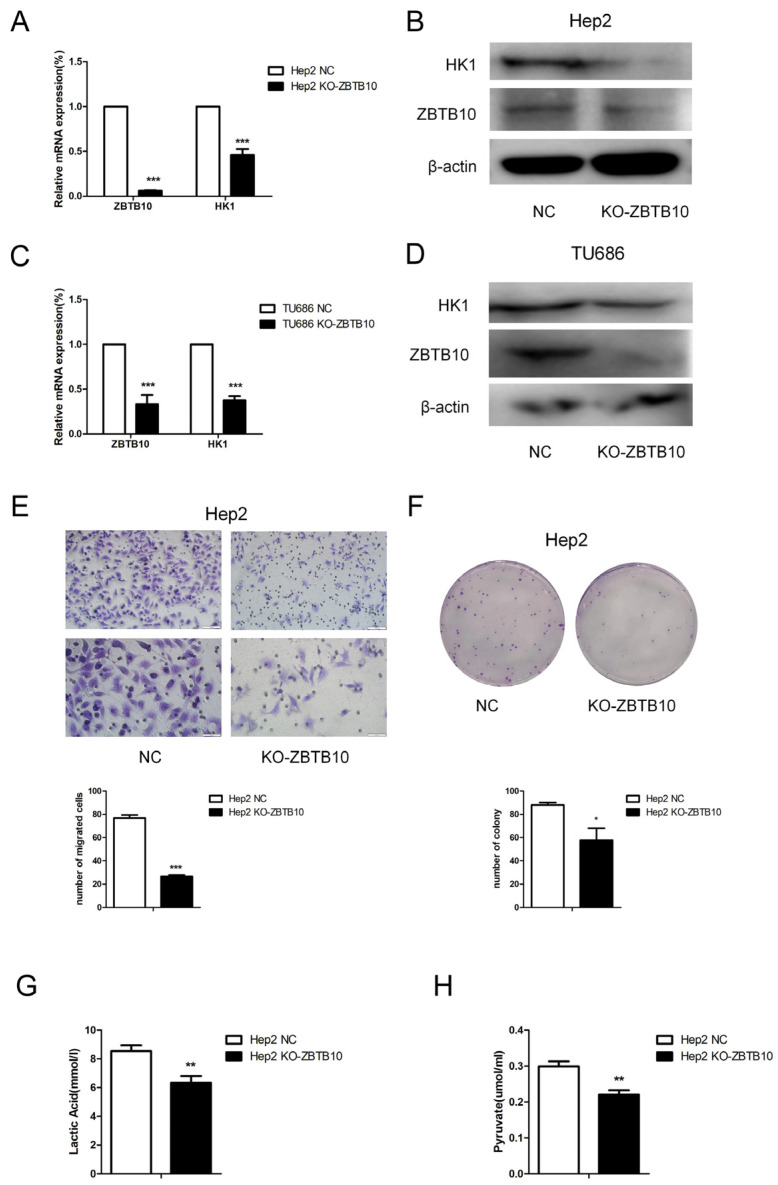
ZBTB10 knockout attenuated glycolysis induced by intermittent hypoxia-exposed M2-type macrophage supernatant. (**A**–**D**) The results of qPCR and Western blotting confirmed ZBTB10 knockout in Hep2 and TU686 cells. (**E**) Transwell migration assay and statistical graph (100× and 200×). (**F**) Colony formation assay and statistical graph. (**G**,**H**) Lactate and pyruvate assays. The data are presented as the mean ± SD values. * *p* < 0.05; ** *p* < 0.01; *** *p* < 0.001.

The above findings suggest that, in laryngeal cancer, the HK1 expression level is positively correlated with that of transcription factor ZBTB10. To confirm that intermittent hypoxia induces the carcinogenic effect of M2 macrophages through ZBTB10, we further investigated the effect of M2 macrophage supernatant on ZBTB10 knockout laryngeal cancer cells. By constructing ZBTB10-sgRNA and knocking out ZBTB10 in Hep2 cells, we found that, after the knockout, both the mRNA and protein levels of HK1 were decreased in laryngeal cancer Hep2 cells (Figure 8A,B). In TU686 cells, the same result was found (Figure 8C,D). After knocking out ZBTB10, the migration and clonogenic capacity of Hep2 cells were reduced (Figure 8E,F). Similarly, the contents of both lactate and pyruvate were reduced in the supernatant (Figure 8G,H). This suggests that knocking out ZBTB10 could reverse the effect of culture supernatant from M2 macrophages exposed to IH on laryngeal cancer cells.

**Figure 9:**
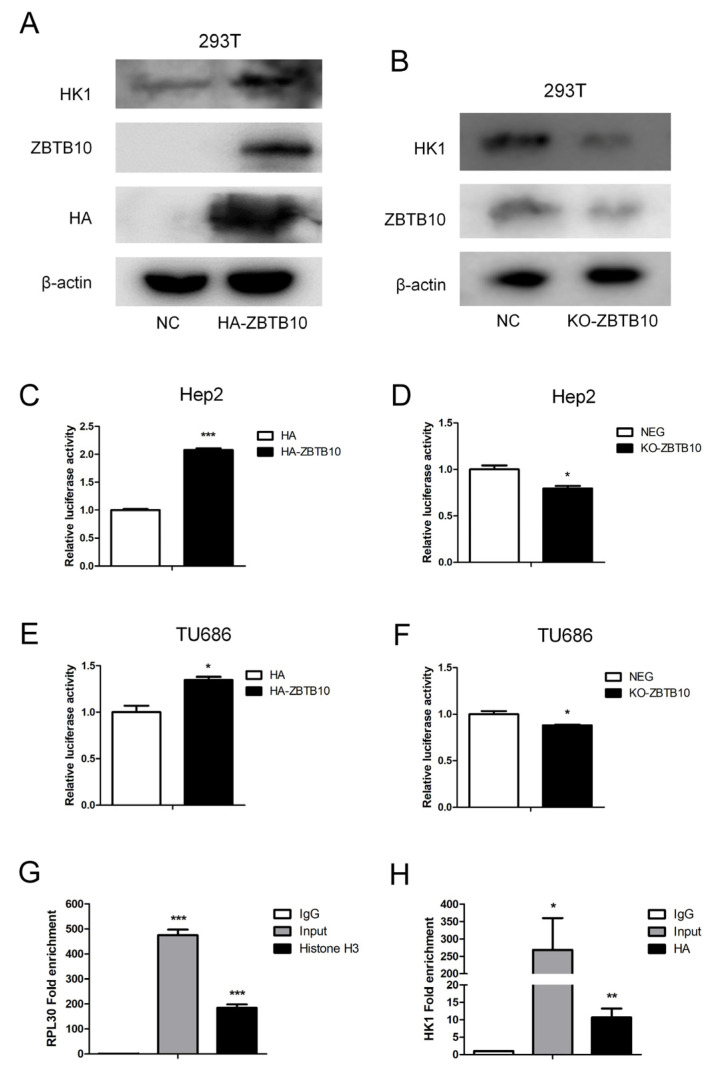
ZBTB10 binds to a candidate promoter sequence in HK1 and transcriptionally regulates HK1 gene expression. (**A**) Representative immunoblot analysis of HK1, ZBTB10 and HA protein expression in ZBTB10-overexpressing cells. (**B**) Representative immunoblot analysis of HK1 and ZBTB10 protein expression in ZBTB10 knockout cells. (**C**) Luciferase activity of HK1 in ZBTB10-overexpressing 293T cells. (**D**) Luciferase activity of HK1 in ZBTB10 knockout 293T cells. (**E**,**F**) Luciferase activity of HK1 in ZBTB10-overexpressing and ZBTB10 knockout Hep2 cells. (**G**) Fold enrichment of the positive control RPL30 in the ChIP assay. (**H**) Fold enrichment of HK1 in the ChIP assay. * *p* < 0.05; ** *p* < 0.01; *** *p* < 0.001.

In order to clarify the regulatory effect of ZBTB10 on HK1, transient transfection of ZBTB10 in 293T cells was performed, which showed that HK1 expression was elevated when ZBTB10 was overexpressed (Figure 9A). When ZBTB10 was completely knocked out, HK1 expression levels decreased (Figure 9B). As ZBTB10 is a transcription factor, to determine whether ZBTB10 plays a regulatory role in HK1, we used the tfTarget database (http://bioinfo.life.hust.edu.cn/hTFtarget#!/prediction, accessed on 10 September 2022) to predict the HK1 promoter sequence and found that there are binding sites for ZBTB10 in the HK1 promoter region (Figure S1). To verify whether ZBTB10 regulates the transcriptional activity of HK1, an HK1 dual luciferase reporter vector was constructed with the HK1 promoter sequence. Transient transfection of 293T cells showed that the luciferase activity of HK1 increased significantly upon transfection of ZBTB10 (Figure 9C), and transient transfection of Flag-HK1 increased the luciferase activity of the HK1 promoter as a positive control (Figure S2), indicating that ZBTB10 overexpression can increase the transcriptional activity of HK1. When ZBTB10 was completely knocked out, the transcriptional activity of HK1 decreased (Figure 9D), indicating that ZBTB10 positively regulates the transcriptional activity of HK1. The same result was found in laryngeal cancer Hep2 cells with transient overexpression or knockout of HK1 (Figure 9E,F). This indicates that ZBTB10 can affect the HK1 promoter region and regulate the transcriptional activity of HK1. To ensure that ZBTB10 transcriptionally regulates the expression of HK1, a chromatin immunoprecipitation (ChIP) assay, which can provide a true and complete indication of the regulatory proteins bound to the DNA sequence and realistically identify the binding of transcription factors to promoters in cells, was conducted. ZBTB10-overexpressing Hep2 cells were chosen, and an anti-HA tag antibody was used for immunoprecipitation. Histone H3 was used as a positive control. We found that the fold enrichment of the positive control RPL30 was 475.08 ± 43.97 and 184.97 ± 25.91 in the input and Histone H3 immunoprecipitation samples, respectively (Figure 9G). The fold enrichment of HK1 was 268.73 ± 183.03 in the input samples and 10.68 ± 5.12 in the HA immunoprecipitation samples (Figure 9H). Hence, our results confirmed that ZBTB10 binds to a candidate sequence in the promoter of HK1 and transcriptionally regulates HK1 gene expression, increasing glycolysis and affecting malignant phenotypes in laryngeal cancer cells.

**Figure 10:**
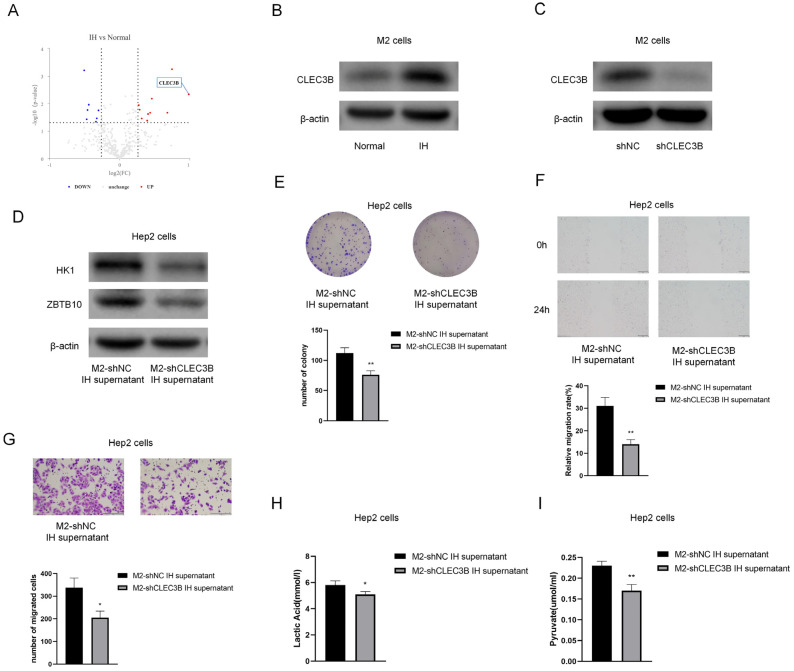
CLEC3B level of M2 supernatant is significantly higher in IH group and showed protumor effect on Hep2 cells. (**A**) Volcano plot displaying proteomics data. (**B**) The results of Western blotting confirmed CLEC3B in M2 cells. (**C**) Representative immunoblot analysis of CLEC3B protein expression in shNC or shCLEC3B M2 cells. (**D**) Representative immunoblot analysis of HK1 and ZBTB10 of Hep2 cells under M2-shNC supernatant or M2-shCLEC3B supernatant treatment. (**E**) Colony formation assay. (**F**) Wound-healing assay (100×). (**G**) Transwell migration assay (100×). (**H**,**I**) Lactate and pyruvate assays. The data are presented as the mean ± SD values. * *p* < 0.05; ** *p* < 0.01.

In order to determine which compound in the M2 culture supernatant causes the effects, a proteomic analysis of the culture supernatant was performed. Figure 10A shows the differently expressed proteins; CLEC3B showed the greatest fold increases in the IH group compared to the normal group. Western blot analysis revealed that the protein expression levels of CLEC3B in M2 cells were elevated, consistent with the proteomic analysis results (Figure 10B). By constructing CLEC3B-shRNA and knocking down CLEC3B in M2 cells (Figure 10C), we found that after knocking down ZBTB10, the protein levels of HK1 and ZBTB10 were decreased in Hep2 cells (Figure 10D). Upon treatment with the M2-shNC supernatant or M2-shCLEC3B supernatant, it was found that CLEC3B knockdown reversed the protumor effect of M2 macrophage supernatant on Hep2 cells (Figure 10E–G). CLEC3B knockdown also reduced the lactate and pyruvate contents in laryngeal cancer cells (Figure 10H,I).

## 3. Discussion

Obstructive sleep apnea (OSA) is a common disease linked to a variety of poor health outcomes. There is striking epidemiological evidence that patients with OSA have higher rates of cancer. There is evidence that OSA is associated with an increased incidence of specific cancers, such as breast, central primary nervous system, nasal, prostate, and colorectal cancers [29]. There generally appears to be a relationship between OSA and cancer, as it has been well established that intermittent hypoxia plays an important role in regulating both tumorigenesis and progression. Therefore, further work is required to elucidate the underlying molecular mechanisms by which intermittent hypoxia promotes tumorigenesis.

Recent studies in vitro and in vivo have shown that intermittent hypoxia could positively modulate tumor development through HIF-1 and miRNA regulation [30,31,32]. It has been reported that intermittent hypoxia promotes lung metastasis of melanoma via oxidative stress and inflammatory responses in vitro [33]. The tumor microenvironment plays an important role in tumorigenesis, and alterations in its characteristics wield substantial influence over both tumor growth and responsiveness to anticancer therapy. A study reported that TAMs from IH-exposed mice markedly increased the proliferation rate and invasiveness of TC1 cells, supporting the idea that IH-induced alterations in TAMs affect tumor promotion [34]. TAMs isolated from IH-exposed mice treated with celecoxib were found to reduce the proliferation of Lewis lung carcinoma LLC1 cells [35], suggesting that targeting TAMs may be a potential strategy for the treatment of cancer in patients with OSA. In our experiment, we found that laryngeal cancer cells exposed to intermittent hypoxia promoted macrophage migration. By inducing M2 macrophages to simulate TAMs, we found that after 24 h of OSA-related intermittent hypoxia, treatment with M2 macrophage supernatant significantly enhanced the malignant behaviors of laryngeal cancer cells, such as growth, proliferation, and migration. Consistent with previous reports, OSA-associated intermittent hypoxia mimics the effects of hypoxia, activating TAMs and promoting malignant transformation of tumor cells. 

The metabolism of tumor cells is different from that of normal cells. One of the characteristics of tumor cells is that they can absorb nutrients extensively, so as to adapt to the changing environment. Even under the condition of sufficient oxygen, they still use glycolysis as the main productivity mode, which is called the Warburg effect [36,37]. High levels of hexokinase in cancer cells are thought to drive glycolysis to maintain aerobic metabolism in cells, which constitutes the Warburg effect [26]. Hexokinase 2 (HK2), a rate-limiting enzyme, exhibits elevated expression levels in neoplastic tissues and is closely related to tumor energy metabolism [38]. In recent years, studies have shown that HK2 in tumor cells not only mediates the Warburg effect but also inhibits tumor cell apoptosis, thereby regulating autophagy and promoting tumor proliferation [39]. Most current research has focused on HK2, while the role of HK1 in tumors has rarely been studied. In our study, differentially expressed genes in the Hep2 laryngeal cancer cells treated with M2 macrophage supernatant were identified by RNA-seq analysis, and the expression of the glycolysis-related gene hexokinase HK1 was significantly increased. Hexokinases (HKs) serve as the inaugural rate-limiting enzymes in the glycolytic pathway, exerting critical control over glucose metabolism in cancer cells [11]. At present, HK2 has been studied widely in tumors. Due to the Warburg effect in tumor cells in the oxygen-replete microenvironment, the rate of glycolysis must be high to generate the large amount of energy required for the rapid proliferation of tumor cells. HK2 is closely related to the Warburg effect in cancer cells [40]. In addition, HK2 can inhibit apoptosis and regulate autophagy in tumor cells [10]. Previous studies have identified several SNPs associated with laryngeal cancer poor prognosis, and HK2 is one of the SNP-mediated oncogene targeted genes [41,42]. However, there are few studies on the regulation of glycolysis by HK1. Oncogene KRAS4A binds directly to HK1 and colocalizes on the outer mitochondrial membrane. KRAS4A enhances the rate of glucose uptake and regulates glucose metabolism in tumor cells by inhibiting the allosteric regulatory effect of HK1 [43]. In our research, we also found that OSA-related IH-induced TAMs promoted glycolysis in laryngeal cancer cells through HK1. The important role of HK1 in TAM-induced glycolysis was verified by knockdown of HK1 in related experiments.

Prediction via the “tfTarget” database (http://bioinfo.life.hust.edu.cn/hTFtarget#!/prediction, accessed on 10 September 2022) suggested that HK1 may be transcriptionally regulated by ZBTB10. We found that the expression of the transcription factor ZBTB10 was also elevated when Hep2 and TU686 cells were exposed to the TAM supernatant. We next confirmed that the expression trend was consistent between ZBTB10 and HK1 in human laryngeal cancer tissues. Moreover, the expression trends of HK1 and ZBTB10 were found to be concordant by transiently silencing or overexpressing ZBTB10 in 293T cells. As it has been reported that ZBTB10 deficiency is related to pyruvate kinase L/R-related metabolic dysfunction [44], we also found that knockdown of ZBTB10 significantly decreased the colony formation and migration abilities of Hep2 cells and that the pyruvate and lactic acid contents decreased when ZBTB10 was deficient. We confirmed the regulation of HK1 transcriptional activity by ZBTB10 in 293T, Hep2, and TU686 cells. 

TAMs are an important part of the tumor microenvironment. Tumor cells use aerobic glycolysis to promote the recruitment of TAMs and regulate the phenotype and related functions of TAMs. TAMs secrete cytokines that play an indispensable role in modulating tumor cell metabolism. Our results showed that the CLEC3B level of the M2 supernatant was significantly higher in the IH group and had a protumor effect on Hep2 cells. CLEC3B encodes tetranectin, a plasminogen kringle-4-binding protein that is located in the cell plasma, extracellular matrix, and exosomes. Tetranectin can induce plasminogen activation, which is associated with tumor invasion and metastasis. It was reported that CLEC3B secreted from cancer-associated fibroblasts promotes colorectal cancer progression [45]. However, another study found that CLEC3B in hepatocellular carcinoma could inhibit metastasis and angiogenesis via AMPK and VEGF signals [46]. More in-depth studies are still needed to clarify the molecular mechanism and function of CLEC3B in cancer progression.

## 4. Materials and Methods

### 4.1. Cell Lines

The human laryngeal cancer cell lines Hep2 and TU686 were used in the study. Hep2 and HEK293T cells obtained from Minzhoubio, Ningbo, China, were cultured with Dulbecco’s modified Eagle’s medium containing 10% FBS. The TU686 cell line, human leukemia monocytic cell line THP1, and murine macrophage cell line RAW 264.7 were cultured with a RPMI-1640 medium containing 10% FBS. Cells were cultured at 37 °C in a humidified atmosphere of 5% CO_2_. M2 macrophages were differentiated from THP1 cells induced by treatment with 100 ng/mL PMA (MCE, Shanghai, China) for 48 h and 20 ng/mL IL-4 (Peprotech, Cranbury, NJ, USA) for 24 h. After the medium was replaced with a fresh medium, the induced M2 macrophages were cultured under intermittent hypoxia conditions (the intermittent hypoxia chambers had oxygen (O_2_) concentrations that oscillated between 5% and 21%, cycling every hour 35 min at 5% O_2_, 25 min at 21% O_2_) for 24 h [44], and the supernatant was collected as a conditional medium for laryngeal cancer cells. All the cell lines were authenticated using short tandem repeat (STR) profiling and routinely tested negative for mycoplasma contamination.

### 4.2. Reagents and Plasmids

The hexokinase inhibitor 2-deoxy-D-glucose (2-DG, HY-13966, MCE, Shanghai, China, −20 °C) was used. To stably silence HK1, a psi-LVRU6GP-scramble shRNA of HK1 was purchased from GeneCopoeia, Inc. (CSHCTR001-1-LVRU6GP, Beijing, China, −20 °C). To verify the expression patterns of ZBTB10 and HK1, the full-length cDNA sequence of ZBTB10 was amplified and inserted into the pEZ-lv201 vector with an HA tag, and the full-length cDNA sequence of HK1 was amplified and inserted into the pEZ-lv242 vector with a Flag tag (GeneCopoeia, EX-T0174-Lv242-B and EX-Z6181-Lv157, Beijing, China, −20 °C). The CRISPR/Cas9 system was used for ZBTB10 interference, and the pLenti-ZBTB10-sgRNA and pLenti-control-sgRNA constructs were purchased from Beyotime (L11170, Shanghai, China, −20 °C). The target sequence for CRISPR interference with the ZBTB10 gene was TCACAGTACCCCTCCTCTGA.

### 4.3. Wound-Healing Assay

A wound-healing assay is typically utilized to quantify cellular migration on two-dimensional surfaces. When the cells formed a confluent monolayer, they were washed with PBS three times, cultured with a serum-free medium, and scratched with 200 μL tips to form a wound. The wound-healing rate was calculated to reflect the cell mobility.

### 4.4. Transwell Migration Assay

Laryngeal cancer cells (5 × 10^4^) were seeded in serum-free medium into the upper compartments of transwell chambers (8 μm pore size; Costar, Sigma-Aldrich, Shanghai, China). Conditioned M2 macrophage culture supernatant (from M2 macrophages cultured in a normoxic or intermittent hypoxic environment) was added to the lower chambers. After incubation for 24 h, noninvaded cells on the upper side of the filter membranes were removed by scraping. Migrated cells on the underside were fixed with 4% paraformaldehyde (Biosharp, BL5391, Hefei, China, room temperature), stained with 0.1% crystal violet solution, and counted under an inverted microscope.

### 4.5. CCK8 Cell Proliferation Assay

Cell proliferation was determined by a CCK8 assay. Hep2 cells (2 × 10^3^) in a volume of 100 μL were seeded into 96-well plates and incubated at 37 °C in a humidified atmosphere of 5% CO_2_ for six days. Cells were cultured with conditioned M2 macrophage culture supernatant (from M2 macrophages cultured in a normoxic or intermittent hypoxic environment), with the medium replenished every three days. Briefly, 10 μL of CCK8 solution (Biosharp, BS350B, Hefei, China, −20 °C) was added to each well, incubated for 1.5 h, and tested once daily. The absorbance at 450 nm was measured using a K3 Touch Microplate Reader (Labserv, Shanghai, China). Each assay was repeated at least three times.

### 4.6. Colony Formation Assay

Cells were plated into six-well plates (500–800 cells/well) and cultured with medium (50% fresh culture medium and 50% conditioned M2 macrophage culture supernatant) for 14 days, with the medium replenished every three days. The colonies formed were then fixed using 4% paraformaldehyde and stained with 0.1% crystal violet solution. The number of clones was determined, and the clone formation rate was calculated. Clone formation rate = (number of clones/number of seeding cells) × 100%. Each assay was repeated three times. 

### 4.7. RNA-seq Analysis

Total RNA was extracted from Hep2 cells that were treated with M2 macrophage culture supernatant, and TRIzol reagent (Invitrogen, 15596026, Waltham, CA, USA, 4 °C) was used to isolate total RNA. For RNA-Seq, library construction and sequencing using a DNBSEQ platform were performed by BGI Company (Shenzhen, China). The expression of genes was quantified as fragments per kb exon per million fragments mapped (FPKM). Differentially expressed gene (DEG) analysis was carried out by DEseq2, with |log2(Fold Change)| > 0 and Padj < 0.05 as the significance criteria [16].

### 4.8. RNA Isolation and Quantitative Real-Time Reverse Transcription Polymerase Chain Reaction (qRT-PCR)

TRIzol reagent was used to isolate total RNA, and PrimeScript RT reagent (TaKaRa, RR037Q, Dalian, China, −20 °C) was used to synthesize cDNA samples. The expression status of the target genes and β-actin were determined by qRT-PCR with ChamQ SYBR Color qPCR Master Mix (Vazyme, Q411-02, Nanjing, China, −20 °C) using a CFX96 Touch Real-Time PCR Detection System. Primer sequences designed for qPCR were as follows: HK1, forward primer 5′-CCAACATTCGTAAGGTCCATTCC-3′ and reverse primer 5′-CCTCGGACTCCATGTGAACATT-3′; ZBTB10, forward primer 5′-AGGAGAGACTGTCCAGCACTT-3′ and reverse primer 5′-GCCGCAAGACATAAGACCAGTAT-3′; and GAPDH, forward primer 5′-GGAGCGAGATCCCTCCAAAAT-3′ and reverse primer 5′- GGCTGTTGTCATACTTCTCATGG-3′. All reactions were run in triplicate.

### 4.9. Western Blot Analysis

Cells were lysed with a RIPA lysis buffer (Beyotime, P0013B, Shanghai, China, −20 °C) containing a protease inhibitor cocktail (Sigma, P8340-1ML, USA, −20 °C) according to the manufacturer’s instructions. Protein concentrations were measured using a Pierce BCA protein assay kit (Beyotime, P0009, Shanghai, China, room temperature). Equal amounts of protein were separated by 10% sodium dodecyl sulfate–polyacrylamide gel electrophoresis (SDS-PAGE) and transferred to a 0.45 μm polyvinylidene fluoride (PVDF) membrane (Merck Millipore, Darmstadt, Germany). The membrane was incubated with diluted primary antibodies against ZBTB10 (1:1000, Elabscience, Wuhan, China), HK1 (1:1000, Proteintech, Hubei, China), CLEC3B(1:1000, ABclonal, Wuhan, China), and β-actin (1:2000, ABclonal, Wuhan, China) at 4 °C overnight. Immunoreactions were then detected with Clarity Western ECL substrate (Biosharp, BL523B, Hefei, China, 4 °C) and visualized with the ECL Detection System (Baygene, Beijing, China). Each assay was repeated three times.

### 4.10. Lactic Acid and Pyruvate Assays

Lactic acid and assay kits and pyruvate assay kits were purchased from Nanjing Jiancheng Bioengineering Institute (Nanjing, China). The procedures were conducted according to the protocol provided by the manufacturer. The absorbance was measured at 505 nm for pyruvate and at 530 nm for lactic acid using a microplate reader. Each assay was repeated at least three times. Each assay was repeated three times.

### 4.11. Tissue Microarray and Immunohistochemistry

Tissue samples of 80 laryngeal cancer patients were collected from Zhongnan Hospital of Wuhan University, and the study was approved by the Institutional Ethics Board of Zhongnan Hospital of Wuhan University. The clinicopathological characteristics of laryngeal cancer patients are listed in Table S1. Paraffin-embedded microarrays of laryngeal cancer tissues were prepared by Wuhan Shuangxuan Biotechnology (Shuangxuan, Wuhan, China). Immunohistochemical staining was performed as previously described [47]. Two pathologists scored the cores, and the tumor expression was compared by the intensity of staining. The score = staining intensity × proportion of positive cells (staining intensity: 0, negative; 1, low positive; 2, positive; 3, high positive; the percentage contribution of positive cells: 1, <10%; 2, 10–35%; 3, 35–70%, 4, >70%).

### 4.12. Luciferase Reporter Assay

The dual luciferase reporter constructs engineered in this study were developed in the pGEX-DL01 vector (GeneCopoeia, Guangzhou, China), which served as the base plasmid. The promoter sequence of the HK1 gene was inserted into the luciferase-expressing reporter vector. Extracts for the dual luciferase reporter assay were prepared using the Dual-Glo^®^ Luciferase Assay System kit (Promega, Madison, WI, USA). Cells plated in 24-well culture plates were mixed with 100 μL of passive lysis buffer (PLB) for 15 min in a shaker with gentle shaking at room temperature. The cell lysate was transferred to a 1.5 mL EP tube. One hundred microliters of LAR II were dispensed into a 1.5 mL EP tube, and up to 20 µL of the cell lysate was carefully transferred into the tube containing LAR II and mixed by pipetting two or three times. The tube was placed in the luminometer, and a reading was initiated. Finally, 100 μL of Stop & Glo^®^ Reagent was added and mixed well by pipetting up and down two to three times, and the Renilla luciferase activity was measured.

### 4.13. Chromatin Immunoprecipitation Assay

A chromatin immunoprecipitation assay kit (SimpleChIP^®^ Plus Sonication Chromatin IP Kit #56383, Cell Signaling Technology, Boston, MA, USA) was utilized according to the manufacturer’s instructions. Cells were incubated in 1% formaldehyde for 10 min at room temperature to crosslink proteins to DNA. Glycine was added to a final concentration of 1%, and the mixture was incubated for 5 min at room temperature and centrifuged at 3000 rpm for 5 min. One milliliter of 1×ChIP Sonication Nuclear Lysis Buffer (adding 1×PIC) was used to resuspend the cell pellet, and nuclear preparation and chromatin fragmentation were performed immediately. Crosslinked chromatin was sonicated into fragments, and then the fragments were immunoprecipitated using different antibodies. The fragments were incubated with an anti-HA tag antibody (MBL, Nagoya, Japan), positive control samples were incubated with an anti-Histone H3 antibody (#4620, Cell Signaling Technology), and negative control samples were incubated with normal rabbit IgG (#2729, Cell Signaling Technology). Samples were incubated at 4 °C with shaking overnight. Then, 30 μL of Protein G Magnetic Beads per sample was added the next day and incubated at 4 °C with shaking for 2 h. The Protein G Magnetic Beads were precipitated on a magnetic separation rack, 150 μL of 1×ChIP Elution Buffer was added to each sample, and chromatin was eluted from the antibody-bound Protein G Magnetic Beads by gentle vortexing for 30 min at 65 °C. Six microliters of 5 M NaCl and 2 μL of proteinase K were used for decrosslinking at 65 °C for 2 h. The DNA was purified using a spin column, and qPCR was applied for DNA analysis. Primers for HK1 promoter analysis were as follows: forward primer 5′-CCTGGATGCCCAAGAGCAAG-3′, reverse primer 5′-TGTATTGCTGAACCCAAAGCG-3′. Each assay was repeated three times.

### 4.14. Statistical Analysis

All the data were presented as means ± standard deviation (Mean ± SD) for at least three independent experiments. Statistical significance was determined by Student’s *t*-test between two groups, and one-way ANOVA when there were more than two groups. Linear regression analysis was used. Statistical analysis of the resulting data was performed using GraphPad Prism 8 software (GraphPad Prism software Inc. San Diego, CA, USA) software. *p* < 0.05 was considered to indicate a significant difference.

## 5. Conclusions

IH promotes TAM-induced glycolysis in laryngeal cancer cells via regulation of HK1 expression through activation of CLEC3B and ZBTB10. Targeting CLEC3B or ZBTB10 may reveal a new therapeutic model in its regulation of hexokinase and its influence on glucose metabolism.

## Data Availability

The other data used to support the findings of this study are available from the corresponding author upon request.

## Supplementary Materials

The following supporting information can be downloaded at: https://www.mdpi.com/article/10.3390/ijms241914808/s1.
