# Supplementary material for: Intermittent Hypoxia Promotes TAM-Induced Glycolysis in Laryngeal Cancer Cells via Regulation of HK1 Expression through Activation of ZBTB10
Source: Int J Mol Sci. 2023 Sep 30;24(19):14808. doi: 10.3390/ijms241914808 (PMC10573418; doi:10.3390/ijms241914808)
Supplement: Supplementary file 1 [file ijms-24-14808-s001.zip › Figure S1.pdf]

TF	Pattern name	Source	Sequence name	Start	Stop	Strand	Score	P value	Q value	Matched motif
ZBTB10	m-dataset-3775-1	hTFtarget	hg38_ncbiRefSeq_NM_000188.3	1252	1267	+	10.0921	8.52e-05	0.168	GAGGGAGAGCCACCGG
ZBTB10	m-dataset-3775-1	hTFtarget	hg38_ncbiRefSeq_NM_000188.3	916	931	-	9.96053	9.14e-05	0.168	TAGACAGGTAAGGAAA
