# Supplementary figures and images for: Intermittent Hypoxia Promotes TAM-Induced Glycolysis in Laryngeal Cancer Cells via Regulation of HK1 Expression through Activation of ZBTB10
Source: Int J Mol Sci. 2023 Sep 30;24(19):14808. doi: 10.3390/ijms241914808 (PMC10573418; doi:10.3390/ijms241914808)

A

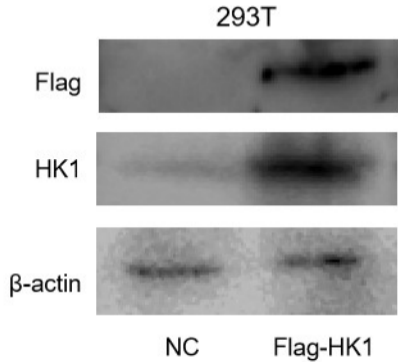

B

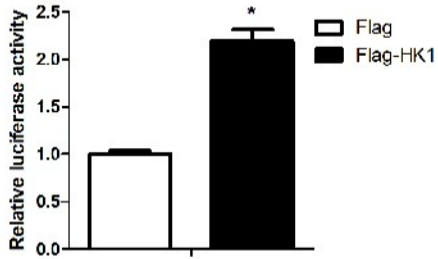

Supplement: Supplementary file 1 [file ijms-24-14808-s001.zip › Figure S2.pdf]
